# Effect of dose rate on pulmonary toxicity in patients with hematolymphoid malignancies undergoing total body irradiation

**DOI:** 10.1186/s13014-018-1116-9

**Published:** 2018-09-18

**Authors:** Dong-Yun Kim, Il Han Kim, Sung-Soo Yoon, Hyoung Jin Kang, Hee Young Shin, Hyun-Cheol Kang

**Affiliations:** 1 Department of Radiation Oncology, Seoul National University College of Medicine, 101 Daehak-ro, Jongno-gu, Seoul, 03080 Republic of Korea; 2 Department of Internal Medicine, Seoul National University College of Medicine, 101 Daehak-ro, Jongno-gu, Seoul, 03080 Republic of Korea; 3 Department of Pediatrics, Seoul National University College of Medicine, 101 Daehak-ro, Jongno-gu, Seoul, 03080 Republic of Korea; 4 Seoul National University Cancer Research Institute, 101 Daehak-ro, Jongno-gu, Seoul, 110-779 Republic of Korea

**Keywords:** Hematolymphoid malignancy, Total body irradiation, Pulmonary toxicity, Dose rate

## Abstract

**Background:**

This study evaluated the effect of radiation dose rate in patients with hematolymphoid malignancies undergoing myeloablative conditioning with total body irradiation (TBI), for hematopoietic stem cell transplantation.

**Methods:**

The incidence of pulmonary toxicity (PT) and treatment efficacy were compared between the conventional (≥ 6 cGy/min) and reduced dose rate (< 6 cGy/min). Seventy-seven patients receiving once-daily TBI between 2000 and 2016 were reviewed. We compared the cumulative rate of PT, overall survival (OS), relapse, and transplantation-related mortality (TRM) between conventional (*n* = 54) and reduced (*n* = 23) groups. Factors associated with PT were assessed in the presence of competing risks.

**Results:**

The median follow-up time was 40.7 months, and PT occurred in 50 patients (64.9%). On multivariate analyses, the groups classified by the dose rate (*P* = 0.010), total dose (*P* = 0.025), and conditioning regimen (*P* = 0.029) were significant factors for the development of PT. OS was significantly reduced when PT occurred (*P* < 0.001). However, the OS, relapse, and TRM were not different between the two groups.

**Conclusions:**

In summary, about two-thirds of the patients undergoing daily TBI experienced PT, which affected OS. Therefore, reducing the dose rate (less than 6 cGy/min) of TBI can decrease the risk of PT, without compromising the treatment efficacy.

## Background

Total body irradiation (TBI)-based myeloablative conditioning, followed by allogeneic stem cell transplantation, is conducted for patients with high-risk or relapsed hematolymphoid malignancies [1–3]. TBI has been used in 46% to 53% of allogeneic hematopoietic stem cell transplantations (HSCT) as a preconditioning component, with other chemotherapeutic agents [4]. There have been a wide variety of TBI doses and fractionation schedules used by each institution. The American College of Radiology (ACR) and the American Society for Radiation Oncology (ASTRO) recommended some basic principles with competing goals of anti-leukemic effects and toxicity reductions; however, a firm consensus has yet to be established [5].

Several complications limit the success of this treatment. Particularly, pulmonary toxicity (PT) is one of the most harmful sequelae of TBI [6–8]. PT is a life-threatening problem after TBI, followed by bone marrow transplantation (BMT), which together, account for 25–50% non-relapse deaths in prior studies [2, 8–13].

In our institution, we have implemented a TBI regimen with once-daily treatment and have gradually reduced the dose rate, to lower lung toxicities. Since 2014, most patients have been treated with a dose rate of less than 6 cGy/min, based on the availability of the extended source-to-surface distance (SSD). The current study examines the risk factors of PT in the once-daily treatment TBI regimen and confirms whether reducing the dose rate affects the clinical outcomes, i.e., overall survival rate, relapse rate, and transplantation-related mortality (TRM), after HSCT. Our goal of the study is to suggest an ideal dose rate of TBI at the once-daily treatment setting.

## Methods

After the approval of the Institutional Review Board, a retrospective analysis of 92 patients (2–63 years old) with hematolymphoid malignancies, who underwent myeloablative TBI as part of the conditioning regimen between 1998 and 2016 at Seoul National University Hospital, was performed. Seventy-seven patients were eligible for this study, except for single fraction TBI (*n* = 9), the presence of existing lung disease (*n* = 3), or failure to perform the planned fraction of TBI due to poor performance status (*n* = 3).

Patients received 9–12 Gy TBI in 3–4 fractions, once-daily, without a day interval. The TBI technique was based on a two-dimensional radiotherapy plan, with 6- or 15-MV photon beam. Patients were positioned in the lateral decubitus position on a stretcher against the wall of the linear accelerator room, to maximize the SSD and field size so that the entire patient was encompassed. Depending on the patient’s height, if the lying position with straightened legs was greater than the field size, the knees posture changed to the bent position. Also, the body thickness of the patient was measured along the entire axis at the prescription point, and thus, the thickness of the compensator at each site was different. In addition to the compensator, a beam spoiler was also used, and both were calibrated as correction factors. The lung shielding technique was not used, but instead, the dose to the anterior lung was attenuated via a lead compensator by 5 to 10% of the prescription dose, and the posterior lung was masked by the arms.

From 1999 to 2013, the dose rate continuously decreased, from a maximum of 17.3 to 6.18 cGy/min, and since 2014, dose rates have been maintained under 6 cGy/min. Our institution has attempted to lower the dose rate of TBI to reduce PT, which has been possible by ensuring an adequate SSD distance (400 cm).

After completing TBI, patients received sequential chemotherapy for conditioning before HSCT. In total, 78.3% of the regimens included chemotherapy, with either cyclophosphamide alone or with one other agent added to cyclophosphamide, such as cytarabine, fludarabine, or etoposide, and the rest used chemotherapy, i.e., cytarabine/fludarabine, busulfan/fludarabine/etoposide, melphalan, melphalan/fludarabine, or thiotepa. There were variations in the pre-HSCT regimen, which was influenced by the type of malignancy, donor source, and prior chemotherapy. Stem cells of the donor source were derived from peripheral blood, bone marrow, or cord blood. Post-transplantation graft-versus-host disease prophylaxis was administered to all patients.

### Definition of PT and other clinical outcomes

According to the criteria commonly used in previous studies [3, 7, 8, 12], PT was defined if patients met any one of the following three definitions, after TBI-based HSCT: (1) two of three of the following clinical symptoms: dyspnea/cough or any reference to respiratory symptoms, pyrexia, and hypoxia/ cyanosis; (2) radiographic evidence: chest radiograph with bilateral diffuse shadowing and/or increased density or increased interstitial markings or opacities, or chest computed tomography with diffuse ground glass opacities; or (3) ventilatory defects, as noted on pulmonary function tests with either restrictive patterns or obstructive patterns. The presence of infection was defined by the detection of infectious agents, through media, such as blood culture or bronchoalveolar lavage. TRM was determined as mortality in the absence of any evidence of recurrence until 3 months after HSCT [14]. In previous literatures, TRM was generally defined as death from any cause without leukemia relapse [15, 16]. Many cases of death after HSCT are due to infectious causes [17], and the risk of opportunistic infection is small after 3 months [18]. For this reason, we limited the definition of TRM to 3 months after TBI followed by HSCT.

#### Statistical analyses

We analyzed factors affecting PT (e.g., group divided by dose rate, total dose (Gy), daily dose (Gy), age at TBI, gender, cancer type, donor type, conditioning regimen, or number of prior chemotherapy regimens) and performed subgroup analyses by the etiology of PT (infection vs. non-infection) in the presence of the competing risks suggested by the Fine–Gray model. After conducting univariate regression of PT, multivariate analysis of PT was done by including covariates in the competing risks regression model with *P* < 0.20. Overall survival, relapse rate, and TRM were examined by the Kaplan-Meier method, with the *p*-value calculated by the log-rank test. All analyses were performed using Stata/MP 14.2 with a significance level of 0.05.

## Results

A total of 77 patients with median age of 19.9 years (range, 1.8–66.4 years) were analyzed for the study. The median follow-up time was 40.7 months. The demographics of the patients are listed in Table 1. We grouped patients by the dose rate of TBI. The dose rate of less than 6 cGy/min was classified as the ‘reduced group (*n*=23)’, and the dose rate of ≥6 cGy/min was categorized as the ‘conventional group (*n*=54)’. Median dose rate was 4.8 cGy/min (range, 4.2–5.2 cGy/min) in the reduced group and 8.6 cGy/min (range, 6.2–17.3 cGy/min) in the conventional group.

**Table 1:** Patient characteristic (*N* = 77)

	Dose rate < 6 cGy/min (*N* = 23)	Dose rate ≥ 6 cGy/min (*N* = 54)	*P*-value
Age at TBI (year)	29.4 ± 18.6 (1.9–66.4)	15.9 ± 8.9 (1.8–41.7)	0.002^b^
Sex			
Male	14 (60.9%)	32 (59.9%)	0.895^a^
Female	9 (39.1%)	22 (40.1%)	
Type of Cancer			
ALL	15 (65.2%)	32 (59.3%)	0.172^a^
AML	2 (8.7%)	14 (25.9%)	
Others	6 (26.1%)	8 (14.8%)	
Total dose (Gy)	11.7 ± 0.9 (9–12)	10.9 ± 1.1 (9–12)	0.004^b^
Daily dose (Gy)	3.0 ± 0.1 (2.5–3.0)	3.1 ± 0.2 (2.3–3.3)	0.001^b^
Daily dose < 3	1 (4.3%)	2 (3.7%)	
Daily dose ≥3	22 (95.7%)	52 (96.3%)	0.017^a^
Donor type			
Related	16 (69.6%)	22 (40.7%)	0.021^a^
Unrelated	7 (30.4%)	32 (59.3%)	
Conditioning regimen			
Cyclophosphamide	4 (17.4%)	8 (14.8%)	0.271^a^
Cyclophosphamide + other^c^	14 (60.9%)	24 (44.4%)	
Others^d^	5 (21.7%)	22 (40.7%)	
Number of prior chemotherapy regimens			
1	3 (13.0%)	14 (25.9%)	0.193^a^
2	8 (34.8%)	23 (42.6%)	
≥ 3	12 (52.2%)	17 (31.5%)	

^a^*P*-value by chi-square test. ^b^*P*-value by Mann-Whitney *U* test

^c^ Other: cytarabine, fludarabine, etoposide

^d^ Others: cytarabine/fludaraibine, busulfan/fludarabine/etoposide, melphalan, melphalan/fludarabine, thiotepa

There was a statistical difference between the groups, according to the dose rate in age at TBI (year), total dose (Gy), daily dose (Gy), and donor cell type. The median follow-up time was 10.6 months (range, 0.7–30.6 months) in the reduced group, and 12.6 months (range, 0.5–191.9 months) in the conventional group; however, the difference between the two groups was not significant. Overall PT, including both infectious and non-infectious types, developed in 50 patients (64.9%). Ten patients (43.5%) in the reduced group and 40 patients (74.1%) in the conventional group had PT, which was significantly different between the groups (*P* = 0.01) (Table 2).

**Table 2:** Incidence of pulmonary toxicity related to time after total body irradiation according to the groups classified by the dose rate

	Reduced group, dose rate < 6 cGy/min (*N* = 23)	Conventional group, dose rate ≥ 6 cGy/min (*N* = 54)	*P*-value
Follow-up time (month)	10.6 (0.7–30.6)	12.6 (0.5–191.9)	0.114^b^
Overall Pulmonary toxicity	10 (43.5%)	40 (74.1%)	0.010^a^
Infectious PT	8 (34.8%)	28 (51.9%)	
Non-infectious PT	2 (8.7%)	12 (21.8%)	
3 month - Pulmonary toxicity	5 (21.7%)	20 (37.1%)	0.085^b^
6 month - Pulmonary toxicity	9 (39.7%)	28 (52.3%)	0.085^b^
Time to PT after TBI (month)	3.7 ± 3.4 (0.5–11.8)	5.2 ± 6.5 (0.1–30.9)	0.085^b^

PT Pulmonary toxicity

^a^*P* by chi-square test

^b^*P* by log-rank test

The median time to onset of PT after TBI was 5.4 months (range, 0.1–30.9 months), and 3.7 and 5.2 months for the reduced and conventional group, respectively, revealing no statistical significance. The incidence of PT during the short-term period of 3 and 6 months after TBI was compared, respectively. The development of PT at 3 and 6 months was 21.7% and 39.7% in the reduced group, and 37.1% and 52.3% in the conventional group, respectively (*P* = 0.085) (Table 2).

Clinical factors associated with PT were analyzed by univariate and multivariate methods, with competing risks regression. On multivariate analysis, overall PT was significantly related to the groups, according to the dose rate (Sub hazard ratio [SHR] 2.61, 95% confidence interval [CI] 1.26–5.40, *P* = 0.010), total dose (Gy) (SHR 1.38, 95% CI 1.04–1.83, *P* = 0.025), and conditioning regimen following TBI (SHR 2.86, 95% CI 1.11–7.38, *P* = 0.029) (Table 3). In subgroup analyses, infectious PT showed a significant association with the groups (SHR 2.38, 95% CI 1.02–5.54, *P* = 0.045), total dose (Gy) (SHR 1.40, 95% CI 1.07–1.85, *P* = 0.025), and daily dose (Gy) (SHR 0.24, 95% CI 0.07–0.83, *P* = 0.025) (Table 4). Also, the groups divided by the dose rate had borderline significance for non-infectious PT (SHR 4.06, 95% CI 0.90–18.28, *P* = 0.068) (Table 4).

**Table 3:** Univariate & multivariate analysis of risk factor for overall pulmonary toxicity

	Univariate analysis			Multivariate analysis		
	SHR	95% CI	*P*-value	SHR	95% CI	*P*-value
Group						
Reduced dose rate group	1 (ref)					
Conventional dose rate group	1.86	0.93–3.71	0.078	2.61	1.26–5.40	0.010
Total dose (Gy)	1.19	0.94–1.51	0.156	1.38	1.04–1.83	0.025
Daily dose (Gy)						
Daily dose < 3	1 (ref)					
Daily dose ≥3	0.67	0.36–1.25	0.205			
Women	1.23	0.70–2.18	0.471	–	–	–
Age at TBI (year)	1.00	0.98–1.02	0.723	–	–	–
Cancer type						
ALL	1 (ref)					
AML	1.47	0.81–2.67	0.210	–	–	–
Others	1.17	0.56–2.44	0.682	–	–	–
Unrelated donor	1.22	0.70–2.10	0.482	–	–	–
Conditioning regimen						
Cyclophosphamide	1 (ref)					
Cy + other^a^	1.92	0.76–4.81	0.165	2.86	1.11–7.38	0.029
Others^b^	1.06	0.40 – 2.81	0.911	1.60	0.54–4.73	0.394
Prior chemotherapy regimens						
1	1 (ref)					
2	0.61	0.29–1.30	0.200	–	–	–
≥ 3	1.32	0.69–2.52	0.409	–	–	–

P-by Fine-Gray

*SHR* Sub-hazard ratios, *ALL* Acute lymphoblastic leukemia, *AML* Acute myeloid leukemia, *GVHD* Graft-versus-host disease

^a^ other: cytarabine, fludarabine, etoposide

^b^ others: cytarabine/fludaraibine, busulfan/fludarabine/etoposide, melphalan, melphalan/fludarabine, thiotepa

**Table 4:** Univariate and multivariate analysis of risk factor for infectious and non-infectious pulmonary toxicity

	Infectious			Non-infectious		
	SHR	95% CI	*P*-value	SHR	95% CI	*P*-value
Univariate analysis						
Group						
Reduced dose rate group	1 (ref)			1 (ref)		
Conventional dose rate group	1.77	0.81–3.85	0.153	4.06	0.90–18.28	0.068
Total dose (Gy)	1.38	1.02–1.87	0.038	0.83	0.55–1.26	0.381
Daily dose (Gy)						
Daily dose < 3	1 (ref)			1 (ref)		
Daily dose ≥3	0.52	0.25–1.10	0.087	0.68	0.37–1.22	0.201
Women	1.22	0.64–2.34	0.545	1.10	0.35–3.44	0.871
Age at TBI (year)	1.00	0.98–1.02	0.854	0.99	0.96–1.02	0.486
Cancer type						
ALL	1 (ref)			1 (ref)		
AML	1.69	0.87–3.31	0.124	1.40	0.30–6.57	0.668
Others	1.22	0.48–3.09	0.671	1.26	0.36–4.44	0.718
Unrelated donor	1.30	0.68–2.48	0.436	0.98	0.34–2.85	0.971
Conditioning regimen						
Cyclophosphamide	1 (ref)			1 (ref)		
Cyclophosphamide + other^*^	1.65	0.65–4.20	0.290	Cannot be calculated	Cannot be calculated	0.921
Others^†^	0.80	0.29–2.19	0.659	Cannot be calculated	Cannot be calculated	0.925
Prior chemotherapy regimens						
1	1 (ref)			1 (ref)		
2	0.60	0.27–1.35	0.219	0.45	0.07–2.97	0.405
≥ 3	1.25	0.60–2.60	0.551	2.20	0.42–11.42	0.349
Multivariate analysis						
Group						
Reduced dose rate group	1 (ref)					
Conventional dose rate group	2.38	1.02–5.54	**0.045**			
Total dose (Gy)	1.40	1.07–1.85	**0.016**			
Daily dose (Gy)	0.24	0.07–0.83	**0.025**			
Cancer type						
ALL	1 (ref)					
AML	1.69	0.83–3.42	0.146			
Others	1.41	0.56–3.52	0.463			

*other: cytarabine, fludarabine, etoposide † others: cytarabine/fludaraibine, busulfan/fludarabine/etoposide, melphalan, melphalan/fludarabine, thiotepa

**Fig. 1:**
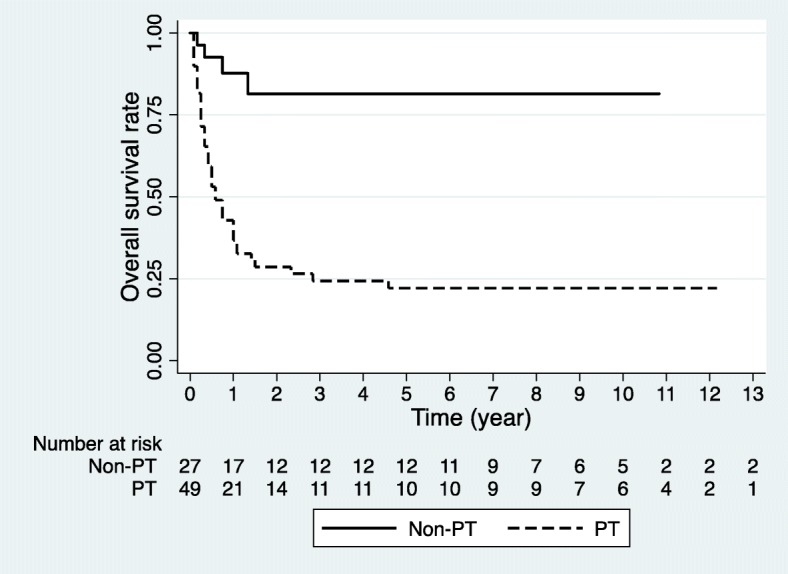
Actuarial overall survival curves in patients with and without pulmonary toxicity (*P* < 0.001 by log-rank test)

**Fig. 2:**
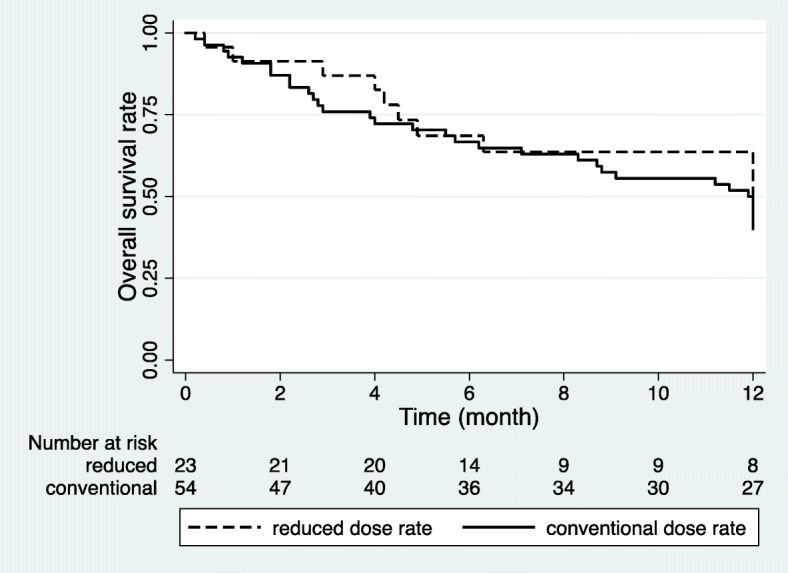
Actuarial survival curves in patients divided by dose rate of total body irradiation. Reduced dose rate < 6 cGy/min and conventional dose rate ≥ 6 cGy/min (*P* = 0.769 by log-rank test)

**Fig. 3:**
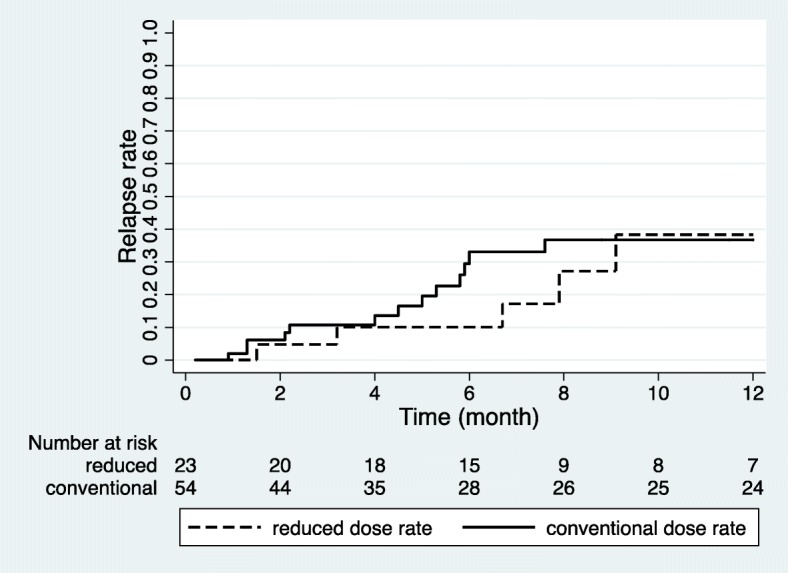
Probability rates of relapse, according to the groups, divided by the dose rate of total body irradiation (*P* = 0.716 by log-rank test)

**Fig. 4:**
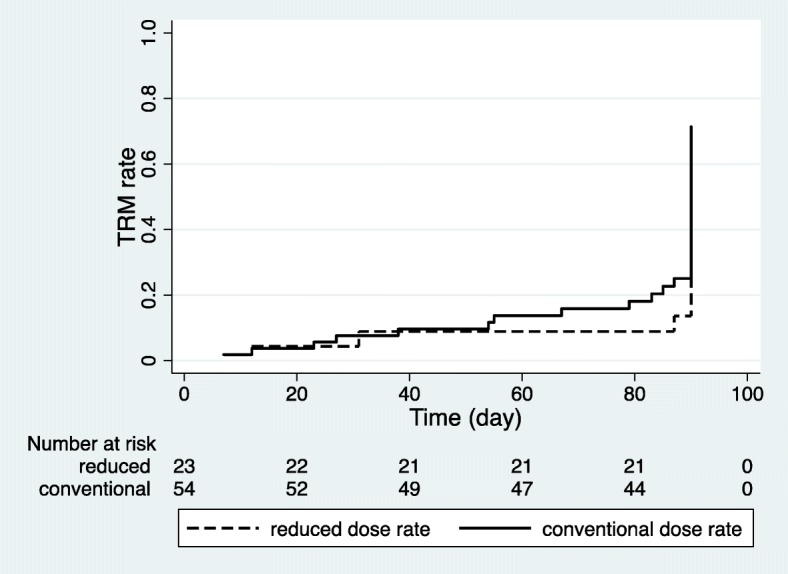
Probability rates of treatment-related mortality, according to the groups, divided by the dose rate of total body irradiation (*P* = 0.131 by log-rank test)

Overall survival was significantly reduced when PT occurred (*P* < 0.001). The 2-year overall survival rate was 28.0% with PT, but 81.5% without PT (Fig. 1). However, the dose rate of TBI did not significantly affect overall survival (*P* = 0.769). Furthermore, other factors, such as total dose, daily dose, and conditioning regimen, did not affect the overall survival in the univariate analysis. The 1-year overall survival rate in the reduced group was 39.8% and 40.7% in the conventional group (Fig. 2). There was no statistically significant difference in the relapse rate between the groups, according to the dose rate. Comparing the 1-year relapse rate after TBI with Fine-Gray model, the reduced group had an occurrence of 41.1%, and in the conventional group, this value was 40.2%, respectively (*P* = 0.953) (Fig. 3). In addition, when analyzing the 3-month TRM after HSCT, no significant difference was found between the groups (*P* = 0.455). The 3-month TRM was 13.0% in the reduced group, and 24.1% in the conventional group (Fig. 4).

## Discussion

PT is a common and severe complication after allogeneic HSCT following TBI, occurring in 25–80% of patients [3, 6, 14, 19–23]. PT after HSCT can develop from either infectious or non-infectious etiologies. PT accounts for approximately 50% of TRM, [3, 6, 19] and many studies have shown that PT leads to a significant reduction in the overall survival rate [3]. Therefore, studies on factors affecting the development of PT have been conducted to reduce lung toxicity, which is detrimental to prognosis after TBI followed by HSCT.

A wide variety of TBI doses and fractionation schedules have been studied in different institutions, to decrease toxicities. Among the factors affecting toxicities during TBI and HSCT, the dose rate of TBI is one of the major components for reducing the development of PT [3, 19, 21]. According to the guidelines published by the ACR and the ASTRO, in 2013, many protocols require a dose rate under 20 cGy/min in the setting of twice-daily treatment [5]. In previously published data, a large retrospective review of 932 patients undergoing TBI followed by HSCT disclosed that a dose rate less than 6 cGy/min was correlated with a decreased incidence of PT [8]. Also, one retrospective study found that using a TBI dose rate of less than 4 cGy/min was significantly associated with reducing the incidence of interstitial pneumonitis [21]. Furthermore, in a mouse model for BMT, a high TBI dose rate was more toxic than a low dose rate, assessed by a lethal dose for 50% mortality and ventilation rate [24]. However, all these studies were the results of TBI settings that were delivered twice-daily.

Although many studies have been based on TBI for twice-daily, it is practically difficult to treat patients for TBI twice a day in the actual clinical settings. For this reason, many institutions have performed TBI on a single fractional day, such as our hospital [5, 14, 19, 25–27]. According to Soejima et al. [15], there was no significant difference in the 5-year overall survival rate and the relapse-free survival between the once-daily TBI regimen and the twice-daily regimen, and even less lung toxicity in the once a day TBI technique. In light of such evidence, our institution has attempted to modify the once-daily TBI protocols to reduce the lung toxicities, by adjusting the total dose, total fraction, dose rate, and SSD, which do not impair the therapeutic effect after HSCT. However, the results of the once-daily based treatment have been scarce, and there are few studies related to clinical outcomes, as well as dose rate and PT, which demands a further investigation. Particularly, since the dose rate is a major factor affecting the development of PT, it is necessary to study the effect of the dose rate on once-a-day treatment and suggest a feasible dose rate that can reduce PT. As mentioned above, guidelines by the ACR and the ASTRO recommended a dose rate under 20 cGy/min in twice-daily regimen, there is no consensus on whether dose rate standard can be applied equally when treating once a day. Furthermore, because twice daily treatment is generally expected to produce less long-term sequelae than once-daily regimen, the criteria for dose rate in once-daily TBI may need to be more stringent than the twice-daily setting.

In the present study, we divided the groups by the TBI dose rate and analyzed the factors affecting lung toxicity in each group. The dose rate was divided by 6 cGy/min as the reference value because our institution has treated all the patients at the lowest possible dose rate since 2014, when extended SSD became available, and most of the TBI dose rates have remained below 6 cGy/min. Our study demonstrated that overall PT and infectious PT were associated with the dose rate of TBI, and may also represent a trend in non-infectious PT. These results suggested that the myeloablative regimen, including TBI before HSCT, caused immunodeficiency and neutropenia that made patients fragile to the opportunistic infections [28].

Although it is well known that reducing the dose rate can lower the incidence of lung injuries [3, 20, 21], it has not been clearly discovered whether or not the reduced dose rate affects the clinical outcomes, such as overall survival, relapse, and TRM after HSCT. Our study revealed that a TBI dose rate < 6 cGy/min did not show statistically significant inferiority in the clinical outcomes as conditioning for HSCT after TBI. These findings would be the basis for treating patients using the once a day TBI technique, with the dose rate under 6 cGy/min, and could be demonstrated if the number of enrolled patients increased.

This study has several limitations. Due to the retrospective nature of our research, it has inherent weak points for inhomogeneity of patient’s characteristics. The sample size is insufficient to confirm the results from the data. Also, there was a possibility to misjudge the etiology of PT, because infectious and non-infectious causes can overlap in certain situations. Nevertheless, the results obtained from this study are meaningful in the actual clinical setting, where there are not many institutions treating with single fraction per day TBI, and the relevant clinical results are limited. Therefore, suggesting an appropriate TBI dose rate to reduce PT will be helpful in treating patients with single fraction per day TBI. Moreover, given the relatively homogeneous total dose and dose per fraction delivered in this study, the effect of the dose rate has increased reliability.

## Conclusions

About two-thirds of the patients undergoing daily TBI for pre-treatment of HSCT experienced PT, which affected overall survival. Reducing the dose rate of TBI decreased the risk of PT, without compromising the treatment efficacy in overall survival, relapse, and TRM. Based on the findings, we suggest reducing the dose rate to less than 6 cGy/min for daily TBI treatment.

## Abbreviations

ACR: American College of Radiology
BMT: Bone marrow transplantation
HSCT: Hematopoietic stem cell transplantation
OS: Overall survival
PT: Pulmonary toxicity
SSD: Source-to-surface distance
TBI: Total body irradiation
TRM: Treatment-related mortality
